# Associations of generalized anxiety and social anxiety symptoms with sleep duration, amount of intense exercise, and excessive internet use among adolescents

**DOI:** 10.1186/s12888-024-06231-y

**Published:** 2024-11-12

**Authors:** Kati Kajastus, Olli Kiviruusu, Mauri Marttunen, Klaus Ranta

**Affiliations:** 1 Faculty of Social Sciences, Tampere University, Tampere, Finland; 2 Mental Health Team, Finnish Institute for Health and Welfare, Helsinki, Finland; 3 Adolescent Psychiatry, University of Helsinki, Helsinki, Finland; 4 HUS Helsinki University Hospital, Helsinki, Finland

**Keywords:** Generalized anxiety, Social anxiety, Adolescents, Sleep duration, Exercise, Excessive internet use

## Abstract

**Background:**

Among adolescents, symptoms of generalized anxiety disorder (GAD) and social anxiety disorder (SAD) are not only clinically significant but also continuingly increasing. During adolescence, individuals begin to establish distinct, self-determined lifestyle patterns. This study seeks to identify the associations between such lifestyle factors and the prevalence of GAD and SAD symptoms.

**Methods:**

The analyzable sample was based on a cross-sectional, nationally representative survey of Finnish 14–18 year old students from comprehensive schools, general upper secondary schools, and vocational institutions. The sample consisted of 60,252 boys and 71,118 girls, with the mean age of 16.05 years (standard deviation 1.06). Symptoms were identified using the GAD-7 (cutpoint of 10), the Mini-SPIN (cutpoint of 6), and the PHQ-2 (cutpoint of 3). Logistic regression models for both GAD and SAD symptoms were stratified by comorbidity group variables.

**Results:**

GAD symptoms were closely related to excessive internet use and decreased sleep duration among adolescents, regardless of comorbidity with depression or SAD. SAD symptoms were associated with excessive internet use and a lack of intense exercise, independent of comorbid depression or GAD.

**Conclusions:**

The findings underscore the importance of considering lifestyle factors when developing interventions to mitigate GAD and SAD symptoms in adolescents. Interventions and policy recommendations aiming at improving adolescents’ health behaviour and mental health should take into account the intertwining of these factors.

## Introduction

Among mental health disorders, anxiety disorders emerge as the most prevalent during the adolescent period [1]. Anxiety symptoms often lead to significant disruption in functioning in important domains of life [2–5]. Among anxiety disorders, generalized anxiety disorder (GAD) and social anxiety disorder (SAD) are both clinically significant and impairing [6, 7]. GAD is characterized by persistent and excessive worry about a variety of everyday topics, which may lead to exhaustion, restlessness and physical symptoms such as muscle tension, whereas SAD is defined by a chronic and overwhelming dread of one or more types of social scenarios [8].

During recent decades, a continuing increase of GAD symptoms has been observed in population studies among adolescents, especially in females [9–11]. Evidence also shows that adolescents’ SAD symptoms have increased lately, especially among girls [12]. This increase of anxiety symptoms occurs during a critical developmental period when adolescents are experiencing multiple developmental changes, and certain self-determined behavioural patterns, such as sleep habits [13] and involvement in internet use are formed [14].

One lifestyle factor that has received increased attention in recent years as a clinical correlate component of GAD and SAD among adolescents is sleep. Among adolescents with anxiety disorders, sleep-related problems are associated with the severity of anxiety [15], and sleep problems during late childhood predict escalating anxiety symptoms in adolescence [16]. Also, deviations in circadian rhythms have been reported to be widely associated with psychiatric problems in general among adolescents [17]. Population research suggests that reduced sleep duration, even without considering sleep quality, may increase the risk of anxiety, even if anxiety does not increase the risk of reduced sleep quantity [18]. Additionally, it’s important to recognize the natural variation in sleep patterns among adolescents, which can contribute to these dynamics [19].

Sleep disturbance is one, although not obligatory, symptom among the six symptoms listed co-occurring with worry for diagnosing GAD [8], and there appears to be a robust association between sleep problems and generalized anxiety in adolescents [20–22]. Studies also show a substantial prevalence of sleep disturbance among adolescents with SAD, although the occurrence is less common compared to adolescents with GAD [21, 23]. In a comparative study, a greater proportion of youth with GAD reported sleep disturbance compared to youth with SAD or any other anxiety disorder [24, 25]. However, as many as 98% of adolescents with GAD and 90% of those with SAD report at least one sleep-related problem [15]. In contrast, some studies have not found a difference between adolescents with and without GAD in sleep-related problems [23].

Physically active adolescents may have significantly lower odds of mood disorder and general psychological distress than less active adolescents [26], and low activity may be related to higher anxiety level [27]. In a Norwegian study decreased physical activity correlated with increased likelihood of a positive SAD screening in adolescents [28]. SAD symptoms are associated with exercise-avoidance motivation [29], not necessarily only because exercise can trigger fear of scrutiny, but also because exercise can trigger weight-related appearance anxiety which manifests as social anxiety [30, 31]. As for GAD, during the COVID-19 epidemic, longer exercise duration per day was negatively associated with GAD symptoms in adolescents [32]. However, in some studies, physical activity has been associated with higher odds of GAD [26]. These differences in results could potentially be attributed to the small sample sizes and research designs that have been assessed to have areas for improvement in studies concerning physical activity and mental health in adolescents [33].

A third lifestyle factor that may contribute to GAD and SAD in adolescents is excessive internet use (EIU). This phenomenon has witnessed an increase in recent years, particularly among adolescent females [34–36]. Based on an umbrella review, associations between general screen use and health-related outcomes, such as depression, show small-to-moderate effects among adolescents [37]. While certain types of screen usage (e.g. excessive television viewing) have been shown to negatively impact health and well-being, the effects of other forms of screen exposure and internet use (e.g. online communication) are not as clearly understood or established, and content seems to play an important part in determining the potential harm or benefit of exposure to screen-based media to adolescents [37, 38]. However, several studies have shown a moderate positive association between problematic internet use and anxiety among youth [14, 39], although not all [40]. Specific to GAD, adolescents’ symptoms seem to be associated with excessive gaming [41], problematic social media use [42], and, at least in psychiatric patients, EIU [43]. Social anxiety seems to predict symptom severity of internet use disorders [44], and internet use for non-communication purposes more social anxiety [45]. However, current research on adolescents’ screen and internet use, being limited in scale, offers inconclusive insights into age-based impacts, highlighting a need for larger studies [37].

When investigating the potential lifestyle factors that may contribute to GAD and SAD in adolescents, the role of comorbidity should be considered. The presence of two or more disorders might interact in ways that exacerbate the effects of each, and comorbidity of anxiety symptoms can significantly impact the symptom severity, functional disability, chronicity, and the amount of treatment, affecting various aspects of adolescents’ lives [46–49]. In adolescents, comorbidity of anxiety disorders occurs very frequently [50], but comorbidity may still be underestimated [48]. Young people with GAD often present with concurrent anxiety disorder or mood disorder [51], depression displaying a high level of co-occurrence [52]. More than two-thirds of adolescents diagnosed with SAD also seem to have one or more comorbid psychiatric disorders [28].

Despite the growing interest in independent associations between sleep duration, physical activity, and internet use with anxiety symptoms, most previous studies have separated these three factors and studied only the relation between a single factor and symptoms of anxiety or depression. However, given the intricate web of connections between lifestyle factors, it is paramount to study these factors in tandem rather than in isolation, also taking into account relevant sociodemographic factors related to anxiety, such as family affluence [53]. The detected connections between the factors include screen time being related to aerobic fitness [54, 55], problematic internet use to a higher risk of sleep disturbance [56], screen time to short sleep duration [57], screen time to exercising less and experiencing disturbed sleep [58], gaming to insomnia [41], and possibly physical activity to sleeping patterns [54, 59]. Therefore, by examining these factors collectively, a more comprehensive understanding of their interrelations and their cumulative impact on mental health can be gained, enabling more effective interventions and holistic approaches to well-being.

The aim of this study was to investigate the associations of the three lifestyle factors with both non-comorbid and comorbid GAD and SAD symptoms in adolescents. More specifically, a large representative sample of Finnish adolescents aged 14 − 18 was considered as we aimed to:

1. estimate and compare the associations of sleep duration, amount of intense exercise (IE), and excessive internet use (EIU) with GAD and SAD symptoms among adolescents, and
2. identify potential modifying roles of comorbid symptoms underlying the above associations.

## Methods

### Participants

The data were obtained from the School Health Promotion (SHP) study, a nationally representative survey that monitors the well-being, health-related behaviour, and schoolwork of Finnish adolescents. The SHP is based on total sampling, and the target groups are pupils in 4th and 5th grades and in 8th and 9th grades of comprehensive school, and 1st and 2nd year students in general upper secondary schools and vocational institutions. The original survey data used in this study, collected in 2021, covered 77% of all Finnish 8th and 9th graders, 65% of all Finnish 1st and 2nd graders from general upper secondary schools, and 35% of all Finnish 1st and 2nd graders from vocational institutions [60]. The SHP survey is conducted every two years by the Finnish Institute of Health and Welfare, and and all schools and institutions with students in the target groups are invited to participate. The SHP covers a range of mental health symptoms and includes validated measures to assess both generalized and social anxiety within the survey. The 2021 sample has previously been used in studies of anxiety symptoms [9, 12] and excessive internet use [35].

Pupils and students filled out the survey anonymously during a school lesson, between March − May 2021, without receiving any financial compensation or other incentives. Responding to the survey was voluntary, and participants gave informed consent by answering the survey. Guardians of participants under 18 years old were informed in advance, and guardians of participants under 15 years old had an option to decline their child’s participation. The study was conducted according to the guidelines of the Declaration of Helsinki. The institutional review board of the Finnish Institute for Health and Welfare has evaluated the School Health Promotion study research plan.

This study included participants aged 14–18 years from comprehensive schools, general upper secondary schools, and vocational institutions. Responses were received from 90,629 8th and 9th graders from comprehensive schools, 40,549 1st and 2nd graders from general upper secondary schools, and 17,122 1st and 2nd graders from vocational institutions. Due to incomplete responses in key variables, 11.42% of the questionnaires were excluded from the present study. Thus, the final analysable sample consisted of 131,370 participants (60,252 boys and 71,118 girls). The mean age (s.d.) of the boys was 16.04 (1.05), and that of the girls was 16.06 (1.06).

### Variables

#### Anxiety measures

Symptoms of GAD were assessed using the seven-item Generalized Anxiety Disorder Scale (GAD-7), a self-report questionnaire designed to identify individuals with probable GAD [61]. The GAD-7 has been psychometrically validated in both adult and adolescent populations [61–63]. The internal consistency of the GAD-7, measured by Cronbach’s alpha, was reported as 0.91 for Finnish adolescents in a previous study [63]. In our sample, it was 0.92, indicating good reliability. Respondents are asked how often they have been bothered by seven core symptoms of GAD during past two weeks, and the responses are scored on a four-point scale as follows: 0 (not at all), 1 (several days), 2 (more than half the days), and 3 (nearly every day). Thus, the total sum score ranges from 0 to 21, and a sum score of 10 represents a moderate or severe level of generalized anxiety symptoms [61]. In the present study, this was used as the cutoff point. All fully completed questionnaires were included. In addition, if only one item response in the GAD-7 was missing, it was replaced by the average of the subject’s responses to the other six questions (*n* = 2,434) and the response was included.

Symptoms of SAD were assessed using the three-item Mini–Social Phobia Inventory (Mini-SPIN; 64). This instrument has been demonstrated to be effective screening measure for adolescent SAD [64], and Cronbach’s alpha for the Finnish translation, based on our data, was 0.88, indicating good reliability. The Mini-SPIN consists of three questions that are rated on a five-point scale as follows: 0 (not at all), 1 (a little bit), 2 (somewhat), 3 (very much), and 4 (extremely). The time interval for reporting symptoms is the past week, and the total sum score ranges from 0 to 12. Items are as follows: [1] “Fear of embarrassment causes me to avoid doing things or speaking to people,” [2] “I avoid activities in which I am the center of attention,” and [3] “Being embarrassed or looking stupid are among my worst fears” [65]. In this study, the cutoff score was set at 6 points, which has been found to be the optimal cutoff score for detecting SAD among adolescents from the general population [64].

#### Depression

The two-item Patient Health Questionnaire-2 (PHQ-2) was used as an indicator of depression. The survey included a depression module with PHQ-2 questions: “Over the last 2 weeks, how often have you been bothered by the following problems [1]? Little interest or pleasure in doing things [2], Feeling down, depressed or hopeless”. For both items, the response options are “not at all”, “several days”, “more than half the days”, and “nearly every day”, scored as 0, 1, 2, and 3, respectively [66]. The PHQ-2 has been found valid for assessing depressive symptoms, with a cutpoint of 3 being optimal for detecting major depression in adolescents [66, 67]. In adult outpatients, the reliability of the PHQ-2, as estimated by Cronbach’s alpha, has previously been reported to be 0.83 [66]. In our data, the corresponding value was 0.83.

#### Lifestyle factors

Sleep. An estimate of the amount of sleep per night was calculated as a weighted average of responses for the question concerning the five weekdays and the question concerning the two weekend days. Sleep amount was estimated as a difference between the questions “At what time do you usually wake up?” (17 time alternatives with 30 min intervals) and “At what time do you usually go to bed?” (19 alternatives). Respondents, who had 7 h or less sleep per night, were classified as having decreased sleep.

Physical exercise. An estimate of the amount of intense exercise (IE) was based on a question “During your leisure time, how many hours per week do you usually engage in physical exercise that causes shortness of breath and sweating?“. Respondents, who had less than one hour of IE per week, were classified as having low IE, as at its very minimum, the recommendation for the amount of vigorous exercise per week for adolescents has been at least 20 min three times a week [68].

Internet use. The five-item Excessive Internet Use Scale (EIU-S), designed to cover five components of behavioural addiction [69], was used to measure problematic internet use experienced by adolescents. The original items comprising the five behavioural addiction symptoms are the following: 1 ) “I have tried unsuccessfully to spend less time on the Internet” (relapse); 2) “I have spent less time than I should have with either family, friends, or doing schoolwork because of the time I spent on the Internet” (conflict); 3) “I have caught myself surfing when I am not really interested” (tolerance); 4) “I have felt bothered when I cannot be on the Internet” (withdrawal symptoms); and 5) “I have gone without eating or sleeping because of the Internet” (salience). The responses are scored on a four-point scale as follows: 1 (never), 2 (not very often), 3 (fairly often), and 4 (very often) [69], and the responses ‘fairly often’ and ‘often’ signify the current presence of a symptom. The scale provides a way of assessing a wide range of potentially problematic internet use behaviours, but does not focus on specific activities, content, or time spent online. The EIU-S has been found to possess good internal consistency in 25 European countries [69], and Cronbach’s alpha for the Finnish translation, based on our data, was .83, indicating good reliability. In this study, an adolescent’s situation was identified as indicative of an increased risk for addictive behavior when the conflict symptom, accompanied by at least three other symptoms, was present [70–72], referred to in this context as ‘excessive internet use’ (EIU).

#### Socioeconomic status

In the survey, the adolescents were asked, on a five-point scale, how they would rate their family’s financial situation. Family’s financial situation was used as a socioeconomic control factor, with responses being scored on a three-point scale (good, moderate, poor).

### Statistical methods

To compare adolescents with pure and comorbid GAD and SAD symptoms, comorbidity groups were formed based on psychopathology (GAD/SAD/depression) inventory scores, with cutpoints described above. In the analyses concerning GAD, the comorbidity categories were “No depression, no SAD”, “Depression only”, “SAD only”, and “Depression and SAD”. When investigating comorbid symptoms associated with SAD, the categories were “No depression, no GAD”, “Depression only”, “GAD only”, and “Depression and GAD”.

Bivariate associations between GAD/SAD symptoms and lifestyle factors were examined using χ^2^ comparisons and univariate logistic regression analyses. Binary logistic regression models were developed with multiple independent variables for both GAD and SAD symptoms, including demographic information. The logistic regression analyses were stratified by the comorbidity group variable due to the high levels of comorbidity and the clinical importance of understanding how these comorbidities might be related to the associations between anxiety symptoms and lifestyle factors. The six independent variables (sex, age group, family’s financial situation, sleep duration, IE, EIU), each of which had a VIF (Variance Inflation Factor) value less than 2, were simultaneously entered into the logistic regression models. The analyses were performed using R 4.2.2. and SPSS version 29.0.0.0.

## Results

In total, 19.6% (*n* = 25,748) of the adolescents reported GAD symptoms and 35.4% (*n* = 46,537) reported SAD symptoms. Univariate analysis results showed that, for GAD symptoms, the three demographic variables (sex, age group, and family’s financial situation) and the three lifestyle factors were all significant at the *p* < 0.001 level (Tables 1 and 2). Positive associations included being female, being older than 15 years old, having a poor or moderate financial situation in the family, sleeping 7 h per night or less, exercising less than 1 h per week, and having EIU. For SAD, associations with all of the above six variables, except age, were significant at the *p* < 0.001 level.

**Table 1:** General characteristics of Finnish adolescents regarding generalized anxiety and social anxiety symptoms (N, %, and Χ^2^ test *p*-value)

Variable		Total		GAD-7 score (≥ 10)			Mini-SPIN score (≥ 6)		
N		%	N	%	*p*-value	N	%	*p*-value	
131 370		100	25 748	19.6		46 537	35.4		
Sex					< 0.001			< 0.001	
Male		60 252	45.9	4 404	7.3		12 854	21.3	
Female		71 118	54.1	21 344	30.0		33 683	47.4	
Age group						< 0.001			0.004
< 15		25 718	19.6	4 504	17.5		9 157	35.6	
≤ 15, < 16		38 828	29.6	7 904	20.4		14 003	36.1	
≤ 16, < 17		34 669	26.4	6 734	19.4		12 162	35.1	
17≥		32 155	24.5	6 606	20.5		11 215	34.9	
Family’s financial situation						< 0.001			< 0.001
Good		95 294	72.5	14 974	15.7		29 712	31.2	
Moderate		29 647	22.6	8 054	27.2		13 291	44.8	
Poor		6 429	4.9	2 720	42.3		3 534	55.0	
Sleep duration, mean per night						< 0.001			< 0.001
At least 7.5 h		111 023	84.5	19 080	17.2		37 126	33.4	
7 h or less		20 347	15.5	6 668	32.8		9 411	46.3	
Intense exercise, hours per week						< 0.001			< 0.001
At least 1 h		111 218	84.7	19 932	17.9		36 472	32.8	
Half an hour or less		20 152	15.3	5 816	28.9		10 065	49.9	
Excessive internet use^a^						< 0.001			< 0.001
No		118 637	90.3	21 059	17.8		39 944	33.7	
Yes		12 733	9.7	4 689	36.8		6 593	51.8	
Comorbidity: depression, SAD						< 0.001			
No depression, no SAD		75 230	57.3	3 120	4.1				
Depression		9 603	7.3	5 164	53.8				
SAD		28 410	21.6	4 320	15.2				
Depression and SAD		18 127	13.8	13 144	72.5				
Comorbidity: depression, GAD									< 0.001
No depression, no GAD		96 200	73.2				24 090	25.0	
Depression		9 422	7.2				4 983	52.9	
GAD		7 440	5.7				4 320	58.1	
Depression and GAD		18 308	13.9				13 144	71.8	

^a^In the EIU-S, the conflict symptom and at least three other symptoms were present

**Table 2:** Results of univariate logistic regression for generalized anxiety and social anxiety symptoms (OR, 95% CI, and Wald test *p*-value)

Variable		GAD-7 score (≥ 10)				Mini-SPIN score (≥ 6)			
		OR	95% CI		*p*-value	OR	95% CI		*p*-value
Sex					< 0.001				< 0.001
Male		1.0				1.0			
Female		5.4	(5.3-	5.6)		3.3	(3.2-	3.4)	
Age group					< 0.001				0.004
< 15		1.0				1.0			
≤ 15. <16		1.2	(1.2-	1.3)		1.0	(1.0-	1.1)	
≤ 16. <17		1.1	(1.1-	1.2)		1.0	(1.0-	1.0)	
17≥		1.2	(1.2-	1.3)		1.0	(0.9-	1.0)	
Family’s financial situation					< 0.001				< 0.001
Good		1.0				1.0			
Moderate		2.0	(1.9-	2.1)		1.8	(1.8-	1.8)	
Poor		3.9	(3.7-	4.1)		2.7	(2.6-	2.8)	
Sleep duration, mean per night					< 0.001				< 0.001
At least 7.5 h		1.0				1.0			
7 h or less		2.3	(2.3-	2.4)		1.7	(1.7-	1.8)	
Intense exercise, hours per week					< 0.001				< 0.001
At least 1 h		1.0				1.0			
Half an hour or less		1.9	(1.8-	1.9)		2.0	(2.0-	2.1)	
Excessive internet use^a^					< 0.001				< 0.001
No		1.0				1.0			
Yes		2.7	(2.6-	2.8)		2.1	(2.0-	2.2)	
Comorbidity: depression, SAD					< 0.001				
No depression, no SAD		1.0							
Depression		26.9	(25.5-	28.4)					
SAD		4.1	(3.9-	4.4)					
Depression and SAD		61.0	(58.1-	64.0)					
Comorbidity: depression, GAD									< 0.001
No depression, no GAD						1.0			
Depression						3.4	(3.2-	3.5)	
GAD						4.1	(3.9-	4.4)	
Depression and GAD						7.6	(7.4-	7.9)	

^a^In the EIU-S, the conflict symptom and at least three other symptoms were present

Additionally, both depression and SAD symptoms had strong positive associations with GAD symptoms. Adolescents with depression symptoms had an odds ratio (OR) of 29.6 compared to those without comorbidities, and adolescents with depression + SAD comorbidity had an OR of 61.0, suggesting a very high GAD + SAD + depression comorbidity in the sample. For SAD, all the comorbidity groups also had a higher odds of symptoms than those with no comorbidities, the highest OR (7.6) being among those with GAD and depression symptoms.

Logistic regression analysis for GAD (Table 3) showed that certain demographic and lifestyle factors were associated with a higher risk of experiencing GAD symptoms even when comorbidities were considered. When participants were stratified by depression and SAD comorbidity, being female and having a poor financial situation in the family both showed a consistent positive association with GAD symptoms. Sleeping 7 h or less per night, and using internet excessively, were also consistently associated with GAD symptoms in all comorbidity groups. GAD symptoms and low IE were somewhat negatively related, but the association was significant only in one comorbidity group: those having comorbid depression symptoms but not SAD symptoms.

The findings from logistic regression analysis for SAD (Table 4) also underscored the increased risk for SAD symptoms for certain groups, even when comorbidities were taken into account. There was a consistent association of SAD symptoms with being female and having a poor financial situation in the family. Adolescents with low level of IE per week, and those with EIU, also showed consistent association with SAD symptoms. These associations persisted across all distinct comorbidity groups. On the contrary, sleeping 7 h or less per night was not significantly related to SAD symptoms across all categories. A moderate relationship was observed between SAD symptoms and sleep, but this was limited to adolescents who had no other symptoms than SAD, and those who had comorbid depression and GAD symptoms at the same time.

**Table 3:** Results of subgroup logistic regressions for generalized anxiety symptoms, stratified by comorbidity symptom groups (OR, 95% CI, and Wald test *p*-value)

Variable		GAD score (≥ 10)															
		No other symptoms				Comorbid depression only				Comorbid SAD only				Comorbid depression + SAD			
		OR	95% CI		*p*-value	OR	95% CI		*p*-value	OR	95% CI		*p*-value	OR	95% CI		*p*-value
Sex																	
Male		1.0				1.0				1.0				1.0			
Female		**6.2**	(5.6-	6.7)	< 0.001	**2.5**	(2.3-	2.8)	< 0.001	**3.8**	(3.4-	4.1)	< 0.001	**2.3**	(2.2-	2.5)	< 0.001
Age group																	
< 15		1.0				1.0				1.0				1.0			
≤ 15, < 16		**1.4**	(1.3-	1.6)	< 0.001	1.2	(1.0-	1.3)	0.007	1.1	(1.0-	1.2)	0.039	1.1	(1.0-	1.2)	0.231
≤ 16, < 17		**1.4**	(1.3-	1.6)	< 0.001	1.1	(1.0-	1.3)	0.060	1.2	(1.0-	1.3)	0.005	1.1	(1.0-	1.2)	0.318
17≥		**1.6**	(1.4-	1.8)	< 0.001	1.1	(1.0-	1.3)	0.056	**1.3**	(1.2-	1.4)	< 0.001	1.1	(0.9-	1.2)	0.333
Family’s financial situation																	
Good		1.0				1.0				1.0				1.0			
Moderate		**1.6**	(1.4-	1.7)	< 0.001	1.1	(1.0-	1.2)	0.058	**1.3**	(1.2-	1.4)	< 0.001	1.1	(1.0-	1.2)	0.021
Poor		**2.5**	(2.1-	2.9)	< 0.001	**1.6**	(1.3-	1.8)	< 0.001	**1.7**	(1.5-	2.0)	< 0.001	**1.6**	(1.4-	1.8)	< 0.001
Sleep duration, mean per night																	
At least 7.5 h		1.0				1.0				1.0				1.0			
7 h or less		**1.7**	(1.6-	1.9)	< 0.001	**1.2**	(1.1-	1.4)	< 0.001	**1.4**	(1.3-	1.5)	< 0.001	**1.3**	(1.2-	1.4)	< 0.001
Intense exercise, hours per week																	
At least 1 h		1.0				1.0				1.0				1.0			
Half an hour or less		1.0	(0.9-	1.2)	0.468	**0.8**	(0.7-	0.9)	< 0.001	1.0	(0.9-	1.1)	0.868	1.0	(0.9-	1.0)	0.214
Excessive internet use^a^																	
No		1.0				1.0				1.0				1.0			
Yes		**1.7**	(1.5-	2.0)	< 0.001	**1.5**	(1.3-	1.7)	< 0.001	**1.6**	(1.4-	1.7)	< 0.001	**1.4**	(1.3-	1.5)	< 0.001

Bold font indicates statistical significance

^a^In the EIU-S, the conflict symptom and at least three other symptoms were present

**Table 4:** Results of subgroup logistic regressions for social anxiety symptoms, stratified by comorbidity symptom groups (OR, 95% CI, and Wald test *p*-value)

**Variable**			**Mini-SPIN score (≥ 6)**															
			**No other symptoms**				**Comorbid depression only**				**Comorbid GAD only**				Comorbid depression + GAD			
			OR	95% CI		*p-value*	OR	95% CI		*p*-value	OR	95% CI		*p*-value	OR	95% CI		*p*-value
Sex																		
Male			1.0				1.0				1.0				1.0			
Female			**2.7**	(2.6-	2.7)	< 0.001	**1.9**	(1.7-	2.0)	< 0.001	**1.6**	(1.4-	1.8)	< 0.001	**1.8**	(1.6-	1.9)	< 0.001
Age group																		
< 15			1.0				1.0				1.0				1.0			
≤ 15, < 16			1.0	(0.9-	1.0)	0.092	1.0	(0.9-	1.1)	0.898	**0.8**	(0.7-	0.9)	< 0.001	0.9	(0.8-	1.0)	0.010
≤ 16, < 17			0.9	(0.9-	1.0)	0.003	0.9	(0.8-	1.0)	0.019	**0.8**	(0.6-	0.9)	< 0.001	**0.8**	(0.7-	0.9)	< 0.001
17≥			**0.9**	(0.9-	0.9)	< 0.001	**0.8**	(0.7-	0.9)	0.004	**0.7**	(0.6-	0.8)	< 0.001	**0.8**	(0.7-	0.8)	< 0.001
Family’s financial situation																		
Good			1.0				1.0				1.0				1.0			
Moderate			**1.5**	(1.4-	1.5)	< 0.001	**1.2**	(1.1-	1.3)	< 0.001	**1.2**	(1.1-	1.4)	< 0.001	**1.2**	(1.1-	1.3)	< 0.001
Poor			**1.8**	(1.7-	2.0)	< 0.001	**1.3**	(1.1-	1.5)	0.003	1.3	(1.0-	1.5)	0.018	**1.3**	(1.2-	1.4)	< 0.001
Sleep duration, mean per night																		
At least 7.5 h			1.0				1.0				1.0				1.0			
7 h or less			**1.3**	(1.2-	1.3)	< 0.001	1.1	(1.0-	1.2)	0.196	1.1	(1.0-	1.2)	0.165	**1.1**	(1.1-	1.2)	< 0.001
Intense exercise, hours per week																		
At least 1 h			1.0				1.0				1.0				1.0			
Half an hour or less			**1.7**	(1.6-	1.7)	< 0.001	**1.4**	(1.3-	1.6)	< 0.001	**1.5**	(1.3-	1.7)	< 0.001	**1.7**	(1.5-	1.8)	< 0.001
Excessive internet use^a^																		
No			1.0				1.0				1.0				1.0			
Yes			**1.5**	(1.4-	1.5)	< 0.001	**1.4**	(1.3-	1.6)	< 0.001	**1.4**	(1.2-	1.6)	< 0.001	**1.3**	(1.2-	1.4)	< 0.001

Bold font indicates statistical significance

^a^In the EIU-S, the conflict symptom and at least three other symptoms were present

## Discussion

This research found that symptoms of GAD and SAD, whether they were pure or comorbid, were associated with several of adolescents’ lifestyle factors in a large, cross-national population sample. A clear association between GAD symptoms and EIU was found, consistent across adolescents who had no comorbidity, or comorbid depression symptoms, SAD symptoms, or both. GAD symptoms were also consistently associated with sleep duration of 7 h or less per night. GAD symptoms did not have a positive association with low IE. SAD symptoms were consistently associated with EIU and low IE, but not with the duration of sleep. The observed associations were evident even when family’s financial situation was controlled for.

The consistency of the results across the comorbidity groups is of particular relevance, as it has been discussed whether psychopathology generally, rather than anxiety specifically, is associated with adolescents’ functional impairment [7]. As symptoms of anxiety and depression are highly correlated and predict each other [73], research of associations between anxiety symptoms and lifestyle factors should take into account different comorbidities. Our study suggests that there is a distinct relationship between certain lifestyle factors and both GAD and SAD symptoms of adolescents, even if especially GAD shares several common symptoms with other anxiety disorders and depression. This is consistent with previous research in adults, suggesting that although GAD has a significant rate of comorbidity with other disorders [74–76], it is a unique condition and should be considered an independent disorder rather than exclusively a residual of other disorders [77, 78].

Since sleeping difficulties may contribute to having a GAD diagnosis [8], it is logical that our results suggest a notable relationship between sleep duration and GAD symptoms, even when other lifestyle variables are controlled for and even when sleep quality per se was not measured. However, this is not consistent with all recent research, as some studies suggest that psychiatric problems, including GAD, are not necessarily associated with sleep duration in adolescents [17]. On the other hand, previous studies with clinical samples have found associations of clinically diagnosed GAD with sleep problems and decreased sleep duration [15, 23, 25]. Some research has found evidence for possible direction of causality, with decreased sleep duration increasing risk for anxiety to a greater degree than anxiety increases risk for reduced sleep [18]. Moreover, fluctuations in sleep duration may have consequences for daytime anxiety [79]. Potential mechanisms include both physiological and psychological processes [22]. Regarding the latter, sleep problems may be indirectly associated with anxiety disorders through e.g. greater suppression of negative emotions and rumination [80].

In our study, adolescents with self-reported EIU had the odds of GAD symptoms 1.7 times as high as those without EIU in the non-comorbid group. In comorbid groups, the respective ORs were 1.6 at highest. For SAD, the respective ORs were 1.5 (non-comorbid) and 1.4 (comorbid). These results are consistent with some previous adolescent studies that also have shown a moderate positive association between problematic internet use and anxiety (Cai et al., 2023). Digital stress, including approval anxiety, fear of missing out, and communication overload, may be mediating, moderating, or both mediating and moderating factors between digital media use and anxiety [81, 82]. The connections may be complex, since anxious adolescents may be more inclined to use the internet to alleviate their anxiety, and, additionally, phenomena associated with EIU may further increase these adolescents’ anxiety [39, 42]. Also, the concept of excessive use is complex and requires a more precise understanding, especially in light of the changing nature of internet accessibility [83]. More research on contextual and activity-related factors, as well as mediation and interaction effects between psychosocial well-being and motivations, is needed in order to achieve a clearer understanding of adolescents’ excessive internet use [84, 85].

We did not find a consistent relationship between the amount of IE and GAD symptoms when comorbidities and other lifestyle behaviours were controlled for. This is inconsistent with previous findings suggesting that infrequent physical activity may be related to higher anxiety symptom levels [27, 32]. However, there has also been discussion whether anxious adolescents engage in IE more times a week than their non-anxious peers, which could be due, for example, to their effort to reduce high levels of anxiety sensitivity or provide physiological resilience to stress [26]. This is consistent with our finding, that for adolescents with depression symptoms, comorbid GAD symptoms were slightly more likely among those who reported at least one hour of IE per week.

According to our results, however, SAD symptoms were consistently associated with low amount of IE among adolescents. This is consistent with the exercise-avoidance motivation that has been detected among adolescents with SAD symptoms, although causal inferences have not been made [29]. Socially anxious young people feel less effective at exercising in public and report lower levels of self-efficacy in such situations [31]. As puberty begins, the adolescent body experiences marked changes in its shape, proportions, and functions, reflecting the process of maturation. Adolescents with SAD symptoms may be especially uncomfortable as visible bodily changes occur during puberty [86]. Avoidance of social situations in which body-weight or appearance may become the center of attention is a central part of appearance avoidance behaviours of adolescents [30]. Considering that exercise often takes place in public contexts, individuals with social anxiety may feel scrutinized when exercising, potentially resulting in the avoidance of IE [29].

This study’s strength is most notably the large nationally representative sample using psychometrically validated instruments. The sample was population based, nationally generalizable, and included more than 60% of all the Finnish adolescents aged 14 to 18 years who were 8th and 9th graders from comprehensive school, 1st and 2nd graders from general upper secondary schools, or 1st and 2nd graders from vocational institutions [60].

While our study presents essential findings, it is not without limitations. First, because of the cross-sectional design, the extent and direction of causality between GAD/SAD symptoms and the perceived lifestyle behaviours remain unknown. Second, the data used were based solely on adolescents’ self-reports, which could lead to potential overestimations or underestimations of symptoms and behaviours. Some adolescents with distorted responses are likely to report extreme levels on psychosocial and behavioural outcome variables [87]. The scales used did not include information about specific internet activities, time spent online, or sleep quality. Also, since many different phenomena were investigated within the same models, the amount of missing observations accumulated when using listwise deletion.

The data were collected during the COVID-19 period, which may have affected sampling, exacerbated symptoms [9, 12], and influenced certain adolescent behaviours such as excessive internet use [34, 35]. Following the pandemic, it will be imperative to pay renewed attention to various aspects, especially those related to social interactions. More research is needed to fully understand the studied associations, and to develop effective interventions for adolescents who struggle with both anxiety and lifestyle behaviours related to the symptoms. It is important to bear in mind that parenting factors may also have an effect on adolescents’ lifestyle factors [88], and on their anxiety levels and social functioning [89]. Further research with a longitudinal design and use of objective measures to assess non-comorbid and comorbid symptoms as well as different lifestyle behaviours should be conducted to understand the causal pathways and directions of the associations. It is also important that future studies take into account more specific details such as sleep quality, specific internet activities, and time spent online.

Understanding the comorbidity of anxiety and mood symptoms in adolescents can inform interventions and treatments that target specific symptoms and improve emotional well-being. Previous research has indicated that cognitive behavioural therapy (CBT) is effective in treating adolescents’ GAD and SAD symptoms [90]. With especially EIU and sleep duration being central in adolescents GAD symptoms, and EIU and reduced IE in SAD symptoms, it is important that the relationship between mental health and lifestyle factors is taken into account in future interventions. There are CBT methods, such as behaviour activation, cognitive restructuring, stress reduction techniques, and exposure therapy, that seem effective for improving the situation of youths with EIU [91] and sleep problems [92, 93]. Assessing adolescents’ digital media use in the context of their psychological and social functioning may be essential in treatment contexts [81]. Physical activity, on the other hand, may have a small beneficial effect for reduced anxiety [33] and may be a promising means for the treatment of SAD [94].

## Conclusions

The current study suggests that EIU and decreased sleep duration are closely related to GAD symptoms among adolescents, regardless of comorbidity with depression or SAD. Adolescents with GAD are usually afraid of uncertainty and terrible things happening to them, and it seems that this tendency is essential in relation to lifestyle factors even when comorbid symptoms do not exist. Also, EIU and decreased IE are related to SAD symptoms among adolescents, regardless of comorbidity with depression or GAD.

Our findings are likely to be especially helpful in the development of targeted interventions seeking to mitigate the negative effects of persistent and excessive worry about a variety of everyday topics, as results indicate that patterns of internet use and sleep may have an important role in enhancing wellbeing among anxious young people. Also, clinicians who are working with adolescents with social anxiety should consider addressing the ways in which their social fears relate to behaviours concerning exercise and internet use. Policy recommendations aiming at improving adolescents’ health behaviour and mental health should take into account the intertwining of these factors.

## Data Availability

The metadata for the SHP datasets are publicly available through the Data Resources Catalogue (www.aineistokatalogi.fi/catalog). While the survey data themselves are not openly accessible, they can be accessed for research purposes through an application processed by the Finnish Social and Health Data Permit Authority (Findata, www.findata.fi/en).

## Abbreviations

GAD: Generalized anxiety disorder
SAD: Social anxiety disorder
EIU: Excessive internet use
IE: Intense exercise
SHP study: The School Health Promotion study
GAD-7: The seven-item Generalized Anxiety Disorder Scale
Mini-SPIN: The three-item Mini–Social Phobia Inventory
PHQ-2: The two-item Patient Health Questionnaire-2
EIU-S: The five-item Excessive Internet Use Scale
VIF: Variance Inflation Factor
OR: Odds ratio
CI: Confidence interval
CBT: Cognitive behavioural therapy
